# Influence of Vamp Opening Configuration on Foot–Footwear Biomechanics: A Finite Element Analysis of Women’s Low-Heeled Court Shoes

**DOI:** 10.3390/bioengineering13080913

**Published:** 2026-08-12

**Authors:** Arina Seul, Aura Mihai, Mariana Costea, Raluca Lupu, Carmen Cornelia Gaidau, Antonela Curteza

**Affiliations:** 1 Faculty of Industrial Design and Business Management, “Gheorghe Asachi” Technical University of Iasi, 700050 Iasi, Romania; arina.seul@academic.tuiasi.ro (A.S.); raluca.lupu@academic.tuiasi.ro (R.L.); antonela.curteza@academic.tuiasi.ro (A.C.); 2 National Research and Development Institute for Textiles and Leather—INCDTP, 030508 Bucharest, Romania; carmen.gaidau@incdtp.ro

**Keywords:** finite element analysis, simulation, footwear, footwear–foot interaction

## Abstract

Footwear geometry plays an important role in determining mechanical performance, plantar load distribution, comfort, and foot stability during gait. Understanding how constructive design parameters influence foot biomechanics is essential for developing footwear that improves comfort while reducing excessive mechanical loading. Although various finite element studies have investigated insole and outsole design, midsole materials, and plantar pressure redistribution, comparatively little attention has been paid to the influence of upper construction parameters, particularly vamp opening configuration, on the biomechanical behaviour of feet and footwear. This study investigates how the opening amplitude of the vamp affects the biomechanical response of three constructive variants—medium (M1), wide (M2), and narrow (M3) vamp openings—developed on a common shoe last derived from anthropometric data. Finite element analysis was conducted using ANSYS 17.2, with 3D models built in Delcam Crispin ShoeMaker Pro 2015 R2 for the three loading scenarios. Total deformation and von Mises stress were extracted as primary output parameters for both the foot and footwear. The results indicate that wider vamp openings increase structural flexibility, with M2 recording the highest total deformation across multiple scenarios, whereas narrower openings generate elevated stress concentrations, particularly in loading scenario 2. The medium vamp opening (M1) demonstrated the most favourable stress distribution overall, with a maximum stress of 1.838 Megapascals (MPa). Validation against experimental plantar pressure data confirmed that loading scenario 3 follows the same plantar pressure distribution trend as in the biomechanical study. The results confirm finite element analysis as an effective computational tool for footwear design evaluation and indicate that vamp amplitude should be considered alongside material selection and geometry as a key variable influencing comfort, fit, and structural performance.

## 1. Introduction

The interaction between the human foot and footwear constitutes a complex biomechanical system whose performance is influenced by the structural configuration of the shoe, the material properties of each component layer, and the loading conditions imposed during locomotion [1]. Accurate evaluation of the mechanical behaviour of footwear is therefore highly relevant to foot and footwear performance.

The geometry of the footwear upper plays a fundamental role in maintaining proper foot function during walking by providing an appropriate balance between structural support and freedom of movement. In addition to securing the foot within the shoe, the upper helps control foot motion throughout the gait cycle by providing dorsal support and distributing contact pressures over the instep and forefoot. Design features such as the vamp opening, quarter topline, and overall upper fit therefore influence dorsal pressure distribution, gait stability, and the mechanical behaviour of the footwear–foot system [2]. Inadequate upper design may generate excessive localised pressures, increase tissue loading, and contribute to discomfort, pain, or pressure-related pathologies, particularly in anatomically sensitive regions. Consequently, understanding how upper design parameters influence foot–shoe interactions is essential for developing footwear that optimises mechanical performance and may improve comfort while reducing the risk of injury.

Among the upper components, the vamp plays a key role in governing the mechanical interaction between the foot and the footwear. During walking, it provides structural restraint to the dorsal surface of the forefoot while allowing sufficient flexibility to accommodate natural foot motion [3]. Altering the vamp opening changes the amount of material surrounding the forefoot and instep, thereby modifying the structural stiffness of the upper and its resistance to deformation. A larger vamp opening is expected to reduce structural support and increase upper flexibility, whereas a smaller opening may provide greater constraint, potentially increasing stress concentrations in both the footwear and the foot. These changes are therefore expected to influence load transfer and deformation patterns throughout the foot–footwear system during the different phases of gait.

Finite Element Analysis (FEA) has become a widely used method for examining the interaction between footwear and the human foot, providing valuable insights into the impact of footwear on the foot. It enables quantitative, non-invasive assessments of deformation, stress distributions, and strains in each structural component of the foot and footwear system under a user-specified load [4].

FEA has been used to evaluate a variety of phenomena related to foot and footwear components, including the internal loading conditions of the human foot, plantar pressure distribution, the shock-absorbing capacity of midsoles and insoles, outsole traction, midsole hardness effect, and heel height effects. Literature reviews highlight that a significant number of studies focus on the bottom part of footwear or on the interaction between the foot, the sole, and the ground [5,6].

Zhu et al. (2023) built a 3D coupled foot–running shoe FEA model using computer tomography imaging data to analyse the effect of midsole hardness on stress and strain on the plantar fascia, finding that increased stiffness decreases the loading of the plantar fascia but increases contact pressures on the outsole progressively [7]. In a related study, Song et al. (2023) [8] created a 3D foot–shoe FEA model to evaluate the effect of the position and thickness of a carbon-fibre plate on the loading of the plantar tissues and the metatarsals during forefoot running. They reported that lower-positioned and thicker carbon-fibre plates promoted a more even distribution of forefoot loading and therefore reduced the loading rate and the risk of metatarsal stress fractures [8]. In another study [9], the authors evaluated curved versus flat carbon-fibre plate configurations and determined that curvature would provide greater forefoot loading reduction during running due to lower loading compared with flat-plate conditions.

The study conducted by Zhang et al. (2024) [10] illustrates the potential to reduce pressure on diabetic patients’ feet through 3D printing shoe midsoles with auxetic re-entrant ‘hexagonal’ lattice features. FEA was performed in ABAQUS using experimental plantar pressure data to validate the numerical results. Two different configurations of an auxetic thermoplastic polyurethane (TPU) midsole were compared to a non-auxetic reference under both walking and running conditions.

Increased efficiency of running was also explored through the use of Taguchi experimental design optimisation, as demonstrated by Yang et al. (2022), who identified optimal combinations of heel cup geometry, insole material, and midsole thickness, noting that the selection of midsole material is the most significant design parameter affecting plantar pressure redistribution during heel landings [11]. Zhou et al. (2024) also utilised FEA to investigate the heel–ground contact angle of the outsole during forefoot running to determine the potential for stress fractures in the metatarsals [12].

The analysis of footwear uppers presents different challenges compared to soles due to the construction of the upper, which primarily consists of multiple layers of anisotropic materials, including leather, synthetic materials, woven and knitted fabrics, and composite materials. The use of simple isotropic elastic models to represent the mechanical properties of the materials used in the upper is insufficient. Ruperez et al. (2008) [13] developed an FEA model to analyse how the upper part of a shoe deforms during gait. The model was developed as a linear-elastic system with triangular shell elements to simulate the distribution of forces across the foot-upper interface [13]. This was the first tool to allow footwear manufacturers to virtually evaluate the functional performance of their products. Later, Ruperez et al. (2012) proposed an orthotropic linear-elastic model for calfskin to account for large deformations and membrane-bending interactions, and validated their model against both leather-lasting damage and shoe-forming tests [14]. Seul et al. (2023) used FEA to compare three complete footwear designs with the same geometry but different upper materials (leather, knit, and mixed construction) under heel-strike, mid-stance, and push-off loading conditions [15]. The study shows how the type of material used on the upper significantly affects stress distribution throughout the entire shoe. Another study [16] evaluated the effects of pattern line placement and stitch number on joint strength, overall shoe performance, and foot performance across three gait phases. The data showed that shoe design can significantly impact both the distribution and magnitude of mechanical factors, lending further support to the use of complete-shoe FEA for making optimal design choices at the design, technical development and manufacturing level.

In terms of foot and shoe model geometry, as presented by Song et al. (2022) in their systematic review [6], the models were mainly developed from high-resolution magnetic resonance imaging. 3D laser scans and Computer-Aided Design (CAD) software were used to reconstruct the external geometry of the foot surface, and CAD was predominantly used to model footwear components. Foot geometry consisted of bones, cartilage, soft tissue, the plantar fascia, and major ligaments, whereas connective structures were frequently modelled as tension-only truss elements rather than fully 3D solid elements, and non-structural features such as shoelaces were frequently excluded. Footwear models ranged from one-component models to complex ones that comprised an upper, insole, midsole, and outsole. Materials were modelled as linearly elastic, whereas the soft tissue and outsole were modelled as hyperelastic. In terms of boundary and loading conditions, the most widely used methodology for modelling foot-plate systems has been to fix the two proximal ends of the talus and calcaneus, as well as the soft tissue. Ground reaction forces are expressed as a percentage of an individual body weight (approximately 50%). Although very few studies have used more specific subject characteristics derived from motion analysis, little consideration has been given to motion characteristics derived from 3D motion analysis. Most studies considered the interactions between the foot, shoe, and ground to be frictional surfaces with coefficients of 0.5 to 0.6.

A summary of reviewed FEA studies in the field of footwear is presented in Table 1.

As shown above, previous research focused mainly on sole components—including midsoles, insoles, outsoles—or on material modelling of footwear uppers. Therefore, this paper covers a research gap as no relevant attention has been paid to the influence of upper constructive geometry, particularly vamp opening configuration, on the biomechanical behaviour of both the foot and footwear. This research gap motivated the present study, which analysed three constructive variations of women’s court shoes that differ in the amplitude of the vamp opening. Each of the three constructive variations were evaluated under three distinct loading scenarios to assess the impact of design lines on footwear performance and on the foot. The first loading scenario included body-weight forces acting as reaction forces, which are used to apply loads to the three footwear types to assess their performance during the heel strike, mid-stance, and push-off phases of walking. The second loading scenario measured how the footwear performs under forces based on data from our previous study on foot biomechanics. The third loading scenario considered the three phases of the gait cycle, the position of the foot on the support surface, and the gravitational loading on the footwear assembly. This approach allowed for the evaluation of the impact of shoe design on the behaviour of both the foot and footwear and the comparison of three distinct loading scenarios.

This paper is organised into several sections. Section 2 details the materials and methods, including the experimental equipment, model development, finite element modelling procedure, and loading scenarios. Section 3 presents the numerical results obtained from the simulations. Section 4 interprets the findings in the context of previous research and experimental validation. Finally, Section 5 concludes the paper by summarising the principal findings, discussing the study limitations, and outlining future research directions.

## 2. Materials and Methods

### 2.1. Equipment

The Infoot USB system (I-Ware Laboratory Co., Ltd., Minoh, Japan), consisting of a three-dimensional scanner and dedicated software, was used to obtain the three-dimensional shape of the foot. The mean values of anthropometric parameters were used to develop an appropriate last using Delcam Crispin Last Maker Pro 2015 R2 (Autodesk Inc., San Francisco, CA, USA). Footwear modelling was performed using the Delcam Crispin Shoe Maker Pro 2015 R2 software application (Autodesk Inc., San Francisco, CA, USA). FEA was performed using Ansys 17.2 Academic Research Mechanical software application (ANSYS Inc., Canonsburg, PA, USA).

### 2.2. Anthropometric Data Source and Participant Demographics

The anthropometric characteristics used as input for last and shoe modelling were originally collected and reported in a previous study by the authors [17]. The study group consisted of 32 healthy female participants, students at the “Gheorghe Asachi” Technical University of Iași, aged 18–30 years, with an average body mass of 52 kg and a height ranging from 153 to 175 cm, wearing EU (French) shoe sizes 36, 37 and 38 (10, 13, and 9 subjects, respectively). None of the participants presented foot disorders or engaged in high-performance sports or other activities that could influence foot morphology. The three-dimensional geometry of both feet was acquired using the Infoot USB 3D foot scanner and processed with Measure 2.8 software (I-Ware Laboratory Co., Ltd., Minoh, Japan). After scanning, anatomical landmarks were verified and corrected when necessary before calculating the anthropometric dimensions. Eight anthropometric parameters were measured, including foot length (L_f_), ball girth circumference (G_b_), foot breadth (B_f_), instep circumference (C_i_), toe I angle (A_tI_), toe V angle (A_tV_), toe I height (H_tI_), and toe V height (H_tV_). To verify the suitability of the dataset, Student’s *t*-test and Fisher’s test were applied to compare the anthropometric measurements of the left and right feet. No statistically significant differences were identified between sides (*p* > 0.05), allowing the mean values to be used for subsequent analyses. The anthropometric data are reproduced in Table 2 to ensure full transparency and enable trace-back verification [17].

### 2.3. Statistical Analysis

Descriptive statistical analyses were performed for each anthropometric parameter (Table 3), including the arithmetic mean ($\bar{x}$), standard deviation (S), sampling dispersion (S^2^), the minimum (x_min_), maximum (x_max_), amplitudes of variation (A), and the coefficient of variation (CV).

**Table 3:** Statistical indicators characterising anthropometric parameters [17].

Descriptive Statistical Indicators	L_f_ (mm)	G_b_ (mm)	B_f_ (mm)	C_i_ (mm)	A_tI_ (°)	A_tV_ (°)	H_tI_ (mm)	H_tV_ (mm)	Foot Size (INFOOT)
$\bar{x}$	236.7	228.6	93.2	222.8	6.9	10.0	20.3	18.1	37.5
x_min_	222.7	208.8	84.5	174.6	−7.6	−0.5	17.4	14.4	36.0
x_max_	249.1	248.3	100.0	253.7	15.3	19.5	23.2	22.3	38.5
A	26.4	39.5	15.5	79.1	22.9	19.9	5.8	8.0	2.5
S	6.1	9.6	3.8	18.1	4.5	4.4	1.4	2.1	0.8
S^2^	36.8	92.4	14.1	328.3	20.2	19.1	1.9	4.4	0.6
CV	2.6	4.2	4.0	8.1	65.6	43.7	6.8	11.5	2.0

To identify the representative foot size, frequency distributions were constructed for each parameter by dividing the observed value range into six class intervals and computing the class centre, absolute frequency, and relative frequency for each interval. The midpoint of the class exhibiting the highest absolute frequency was considered representative of the analysed population [17]. The value distribution for L_f_ and G_b_ are centralised in Table 4 and Table 5. The resulting representative foot corresponding to French shoe size 37 (240 mm), together with the corresponding mean ball girth circumference (225.2 mm), was subsequently used to develop the shoe last in Delcam Crispin LastMaker Pro 2015 R2 (Autodesk Inc., San Francisco, CA, USA).

### 2.4. 3D Footwear Design and Definition of Model Variants

A classic low-heeled court shoe was designed and modelled using Delcam Crispin ShoeMaker Pro 2015 R2 software (Autodesk Inc., San Francisco, CA, USA). The aesthetic and functional profiles of this footwear type are influenced by the position of the quarter topline and the amplitude of the vamp opening. From a biomechanical perspective, both constructive parameters must be designed to ensure that the footwear fulfils its protective and functional requirements. The vamp line should not traumatise the dorsal surface of the foot, whereas the quarter topline must provide sufficient coverage of the phalangeal region to maintain proper foot stability during gait [18].

The amplitude of the vamp opening and the position of the quarter topline depend on the chosen design, the heel height, and the upper material.

**Figure 1:**
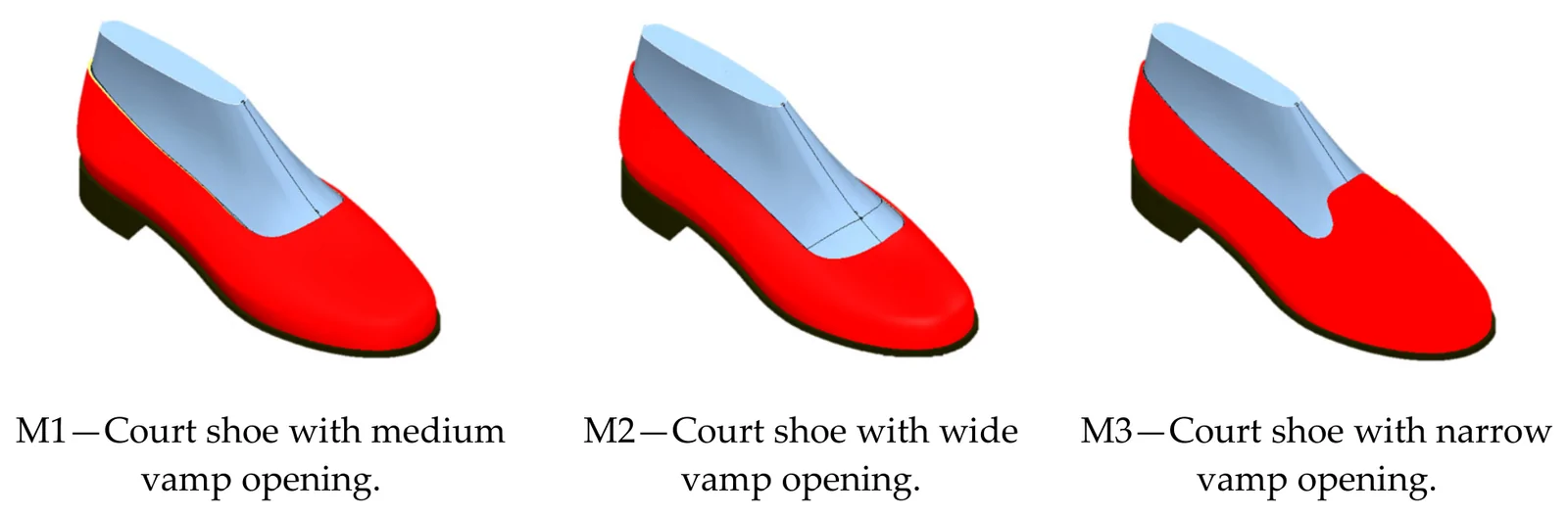
Court shoes—3D models.

Starting from a basic court shoe model (M1), two other models were designed, one with a wide vamp opening (20 mm from the toe line), and another with a narrow vamp opening (slipper-type shoe). All 3 final model variants are shown in Figure 1. The three footwear configurations were selected to represent relevant variations in vamp opening, consistent with footwear design practice [18]. Maintaining identical shoe last geometry, heel height, material properties, and outsole construction isolates the influence of vamp opening amplitude on the mechanical behaviour of the footwear–foot system by ensuring that all other design parameters remain unchanged [13,14,15,16].

### 2.5. Materials

For the analysis carried out in this paper, a new database has been defined (Table 6). The behaviour of the materials was characterised using two constants: Young’s modulus and Poisson’s ratio because they constitute the fundamental elastic parameters required for static FEA and have been widely adopted in previous studies [3,15]. All materials were assumed to show elasto-plastic, isotropic, and homogeneous mechanical behaviour. The foot model used in FEA reproduces the external geometry of a representative female foot. It was generated by modifying a digital shoe last according to mean anthropometric measurements and three-dimensional foot-scanning data obtained from the study population, as described in the previous subsection. Consequently, the model reproduces the external morphology of the foot relevant for footwear design while maintaining compatibility with footwear CAD development. Internal anatomical structures, including individual bones, muscles, ligaments, cartilage, and plantar soft tissues, were not modelled explicitly. Instead, the foot was represented as a homogeneous solid whose external geometry corresponded to the representative anthropometric foot shape. The mechanical properties of the musculoskeletal system—including muscles, cartilage, and joints—were not individually represented in the model [19]. This simplification was adopted because the objective of the present study was to compare the relative influence of different footwear upper geometries rather than to reproduce subject-specific internal tissue mechanics. Similar simplified foot representations have been widely adopted in footwear finite element studies investigating footwear design parameters [6,19]. Muscle activation patterns and individual musculoskeletal properties were not represented; therefore, internal load redistribution associated with muscular activity could not be simulated. The upper assembly was assigned the material properties of bovine leather with a thickness of 1.2 mm, consistent with materials widely used in the footwear industry [20]. The outsole was characterised using rubber material properties [21], whereas the insole was characterised using polyurethane (PU) properties [22], as these materials are widely used in the manufacture of women’s court shoes and are representative of conventional footwear constructions. The same material properties were assigned to all three footwear configurations to ensure that differences in mechanical response originated exclusively from changes in vamp opening geometry rather than from differences in material behaviour.

### 2.6. Setting up the Analysis Conditions

The analysis of the behaviour of the three footwear models was carried out in the Static Structural module of Ansys 17.2 Academic Research Mechanical software (ANSYS Inc., Canonsburg, PA, USA). Materials with defined properties in the previous step were assigned to each geometrical component. The thickness of the upper components was set to 2 mm.

The finite element discretisation was performed using three-dimensional tetrahedral solid elements, which are widely employed for modelling complex geometries because of their ability to accurately represent irregular anatomical and footwear structures. An automatic meshing procedure was applied to all model components using a coarse element size with average node smoothing. The resulting finite element model consisted of 30,208 nodes and 7861 elements (M1), 30,145 nodes and 7787 elements (M2), 30,337 nodes and 7978 elements (M3). The mesh was automatically refined by ANSYS Workbench 17.2 according to the local geometric complexity, providing an appropriate balance between computational efficiency and solution accuracy.

A bonded contact with a contact tolerance of 0.76 mm was defined between the individual upper components and between the upper and the bottom assembly, preventing relative motion at these interfaces. Frictional surface-to-surface contact with a coefficient of friction of 0.6 was assigned between the foot model and the footwear, as well as between the outsole and the support surface, to simulate realistic contact interactions during loading.

Boundary conditions were applied by constraining the lateral edges of the sole and insole in the X-, Y-, and Z-directions. In addition, a fixed support was applied to the superior surface of the foot model to ensure numerical stability and to represent the constraint imposed by the lower leg during the quasi-static loading simulations.

Regarding loading conditions and force distributions, the analysis was conducted for three distinct scenarios. The first scenario represents a simplified engineering approach based on body-weight loading. The second incorporates experimentally measured plantar force distributions, whereas the third additionally considers the angular position of the foot during the gait cycle.

#### 2.6.1. Loading Scenario 1

Based on biomechanical research [23,24], it was considered that in the heel strike and push-off phase, the body acts with a force equal to 1.2∗G, where G is the weight force, given by relation 1.

(1) G = m∗g

where

G—weight force (N);

m—body mass (kg);

g—gravitational acceleration (m/s^2^).

Given that the subjects’ average body mass is 52 kg, a force of 612 N is acting. Since the analysis is performed on one leg, the force is equal to G/2, and the applied force is 255 N.

**Figure 2:**
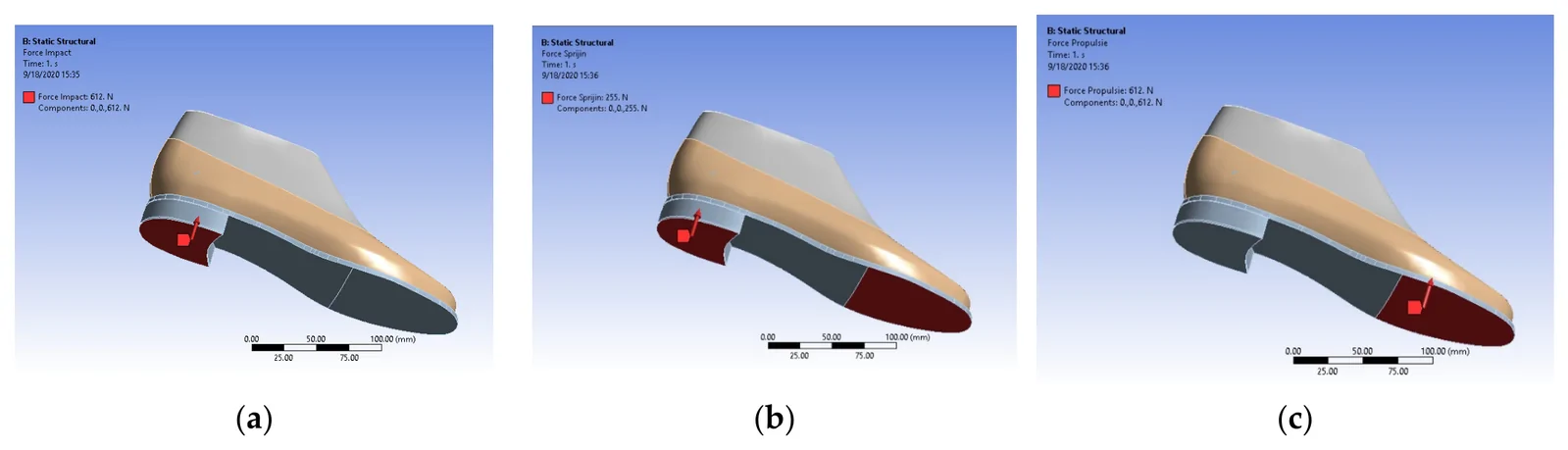
Distribution of the force application areas on the plantar surface of the sole during the heel strike (**a**), stance (**b**) and push-off (**c**) phases.

The forces were applied as ground reaction forces acting on the plantar surface of the sole, simulating the mechanical response of the support surface on the footwear assembly (Figure 2).

The force application areas were defined according to the three anatomical plantar zones—anterior, medial, and posterior—as established in the literature [25]. The anterior zone extends over 0.42 of the total plantar footprint length; the medial zone follows, ending at 0.69 of the plantar footprint length, beyond which the posterior zone begins. Accordingly, during the heel strike phase, the force was applied exclusively to the posterior zone, during the push-off phase to the anterior zone, and during mid-stance across both the posterior and anterior zones.

In this assumption, the angular position of the foot relative to the support was not taken into account.

#### 2.6.2. Loading Scenario 2

For the second loading scenario, the mean force values for dynamic gait conditions were derived from the plantar pressure distribution analysis conducted during normal walking in previous research [26], as shown in Table 7.

**Figure 3:**
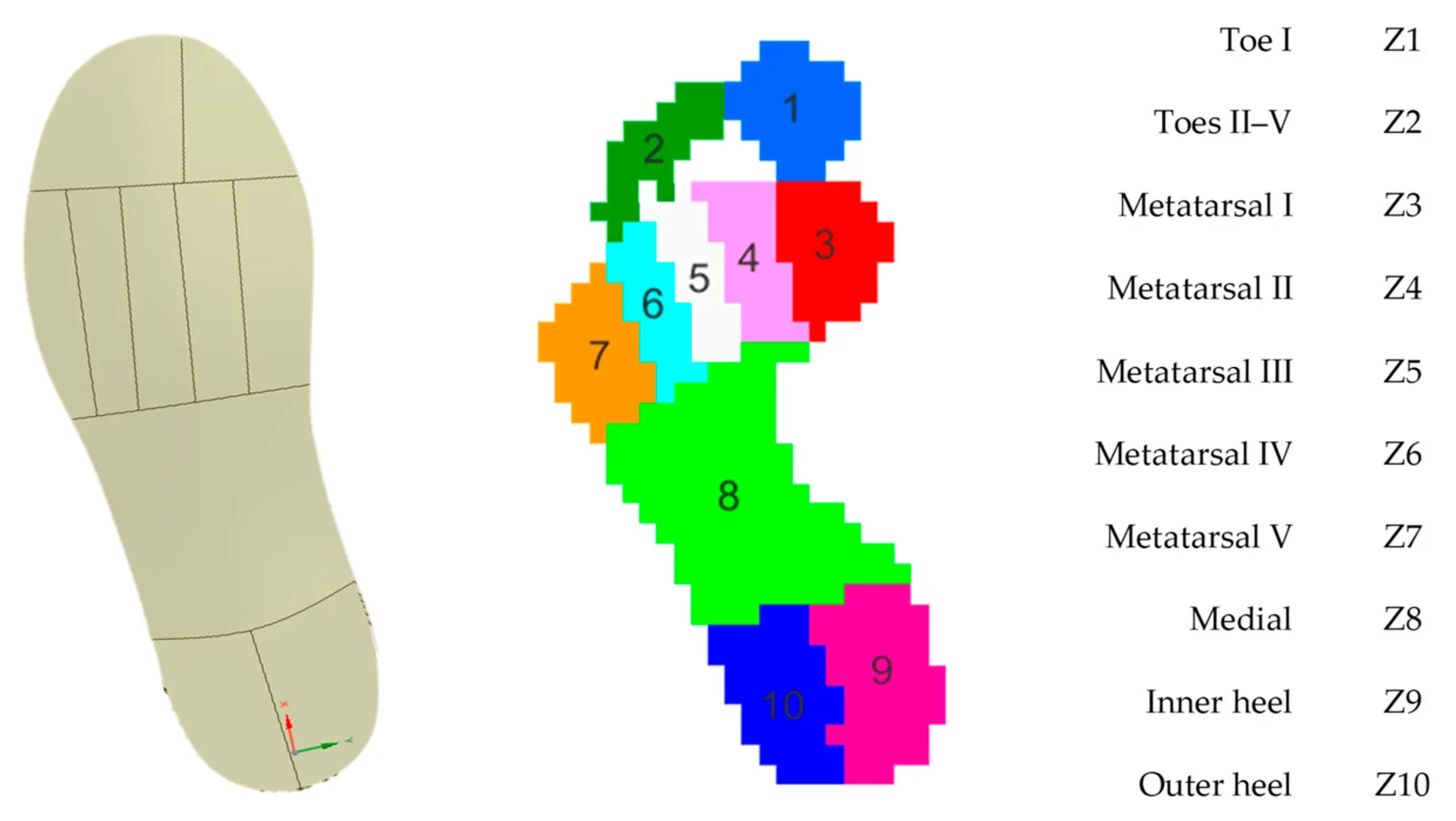
Correspondence between the ten plantar pressure zones and the geometrical subdivisions of the outsole surface.

**Figure 4:**
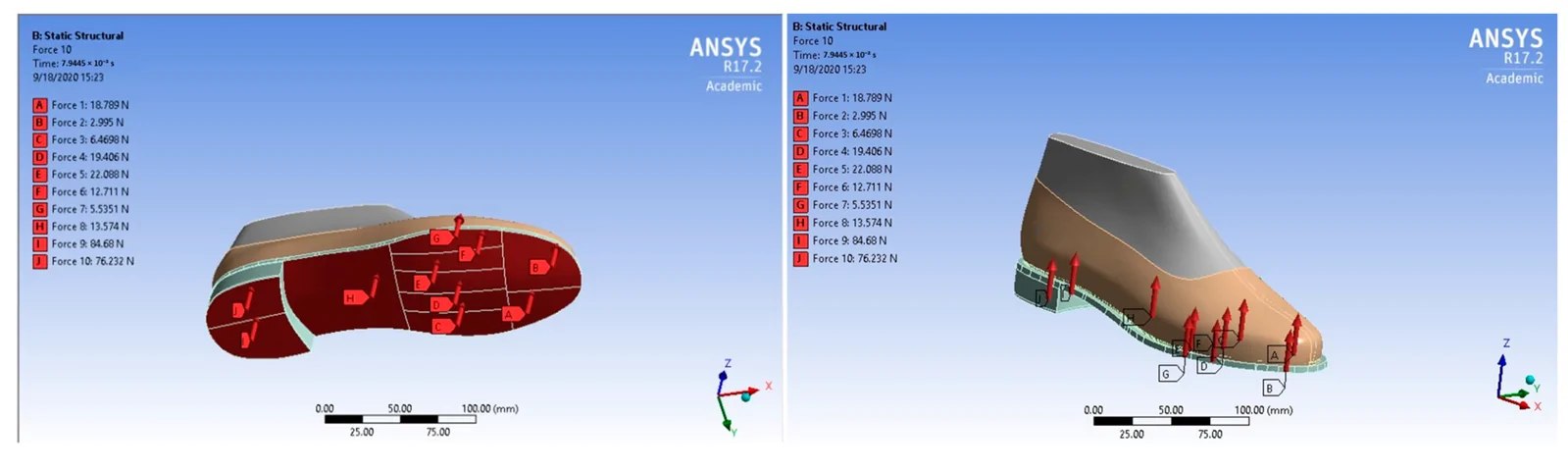
Distribution of ground reaction forces across the ten plantar zones defined on the sole surface.

Similar to the first loading model, the forces were applied as ground reaction forces acting on the plantar surface of the sole. In this model, the application of force was differentiated across ten zones (Figure 3) on the sole surface, in accordance with the plantar pressure distribution model (Figure 4), with each zone assigned an experimentally determined contact time.

#### 2.6.3. Loading Scenario 3

**Figure 5:**
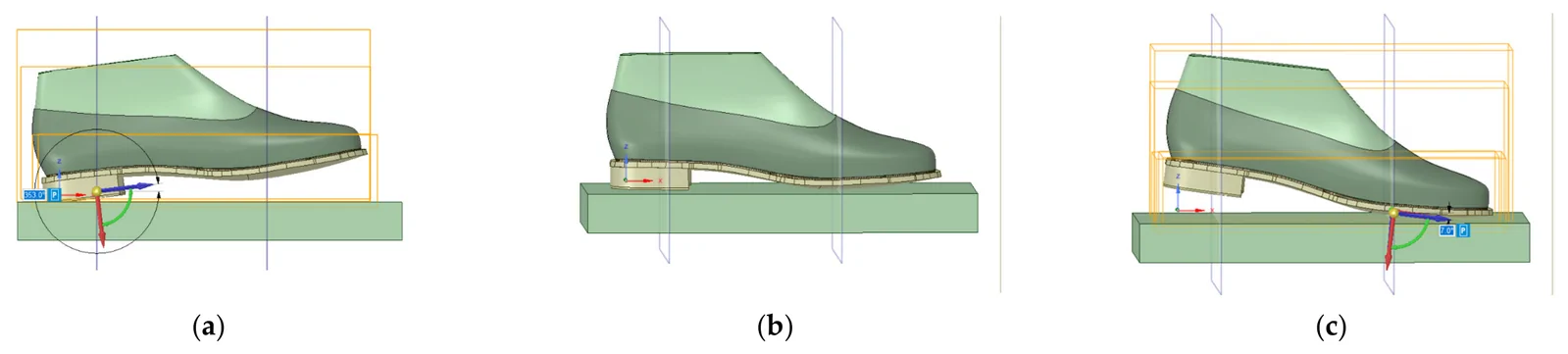
Representation of foot positions in heel strike (**a**), stance (**b**) and push-off (**c**) phases.

The third situation considers the three gait phases—heel strike, stance and push-off. Unlike the first loading model, the angular position of the foot relative to the ground plane is explicitly incorporated. In the stance phase, the foot is in contact with the ground plane over the entire plantar surface, whereas in the heel strike and push-off phases, the foot touches the ground plane partially and is inclined by −7 and 7 degrees [27]. For the heel strike phase, the pivot point was positioned at the mid-heel, corresponding to 0.18 of the total foot length. For the push-off phase, the pivot point was located at the centre of the metatarsophalangeal joints, positioned at 0.72 of the total foot length. The angular position of the foot relative to the ground plane across the three gait phases is illustrated in Figure 5.

The magnitudes of the forces for each gait phase were calculated using the relationships established in the first loading scenario. The resulting forces were applied to the upper surface of the foot in the vertically downward direction, following a loading and boundary condition configuration reported in the literature [28,29]. Displacement of the foot and footwear assembly was permitted along the *Z*-axis, whereas the ground contact surface was modelled as fully fixed. The analysis duration was set to one second. Frictional forces acting at the sole–ground interface and the self-weight of the footwear were considered negligible and were excluded from the analysis.

### 2.7. Configuring Analysis Parameters

The resultant deformation field and the von Mises stress field on both the foot and footwear were evaluated to study the influence of vamp opening amplitude on the mechanical behaviour of the footwear assembly and its impact on the foot under the presented scenarios prior to loading.

The resultant deformation field quantifies the total displacement of the assembly in millimetres (mm), indicating overall structural compliance under loading. The von Mises stress field characterises the stress distribution across the assembly in Megapascals (MPa).

Given the simplified foot geometry, the computed stresses should be interpreted as comparative indicators of the mechanical response of the footwear–foot system rather than as exact physiological stress values within individual anatomical structures.

## 3. Results

The maximum values for the resultant deformation field and von Mises stress field obtained from the analysis are centralised and presented in Table 8 and Table 9 for loading scenario 1, Table 10 and Table 11 for loading scenario 2, and Table 12 and Table 13 for loading scenario 3.

**Figure 6:**
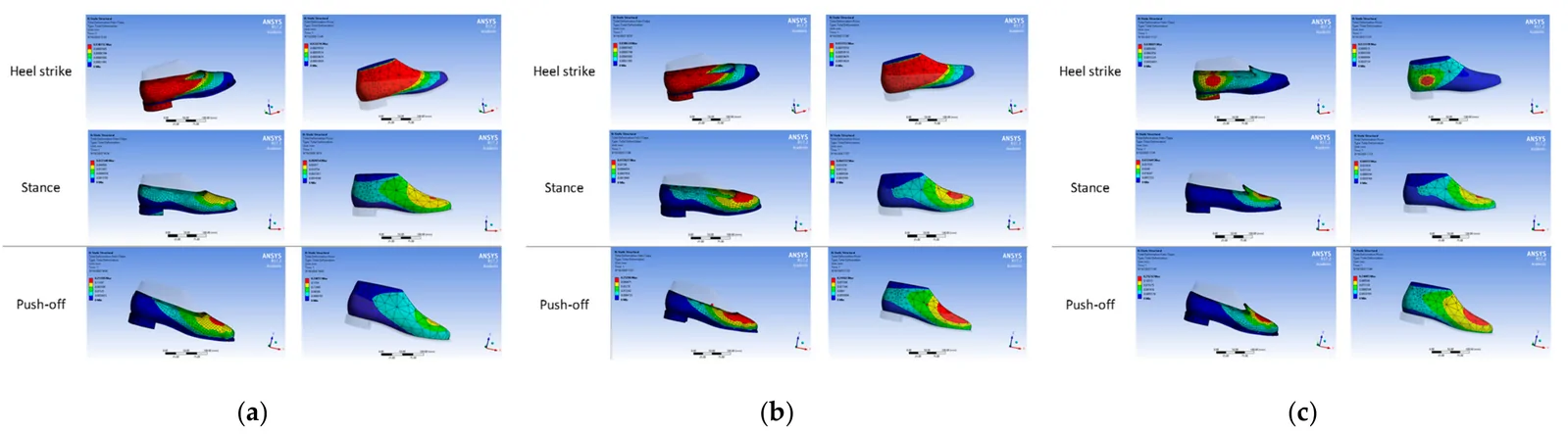
Resultant deformation distribution under loading scenario 1 for (**a**) M1, (**b**) M2, and (**c**) M3.

**Figure 7:**
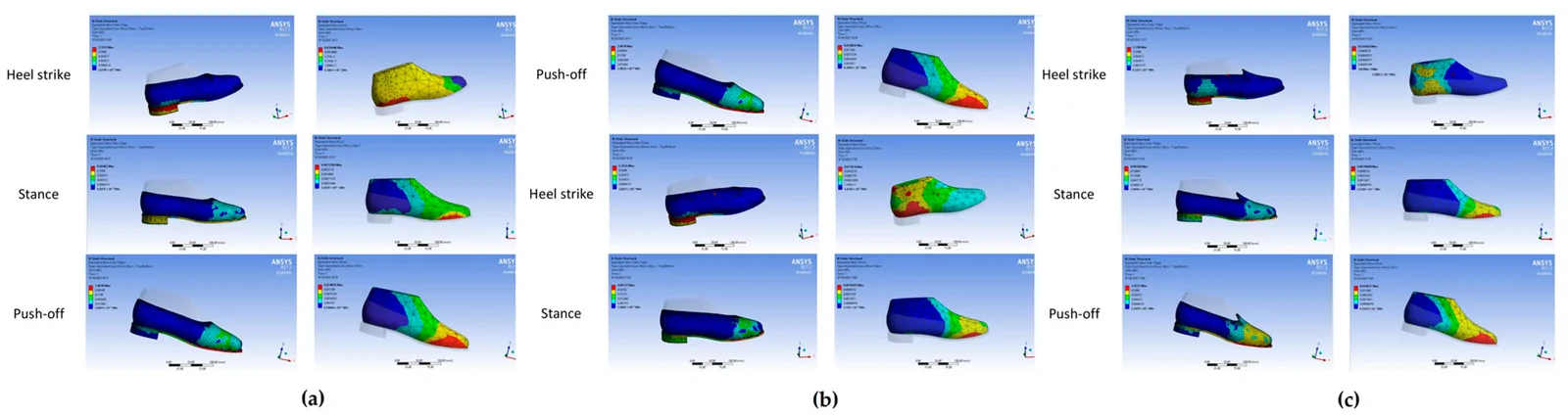
Von Mises stress distribution under loading scenario 1 for (**a**) M1, (**b**) M2, and (**c**) M3.

**Figure 8:**
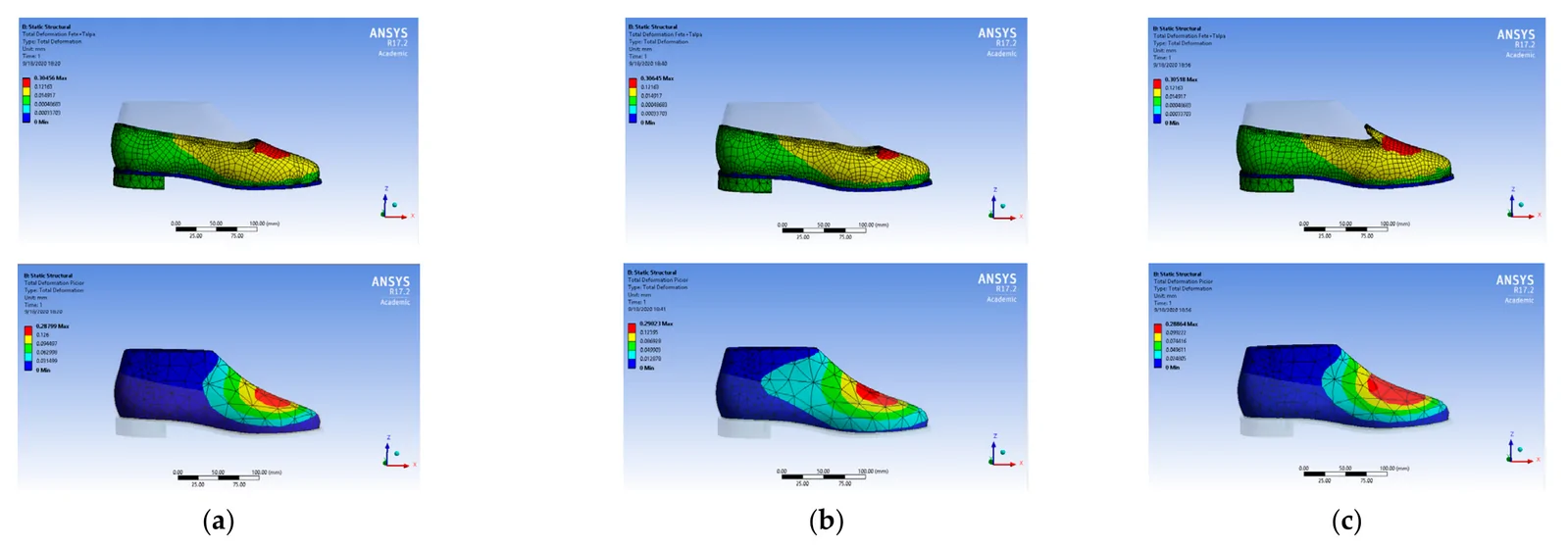
Deformation distribution under loading scenario 2 for (**a**) M1, (**b**) M2, and (**c**) M3.

**Figure 9:**
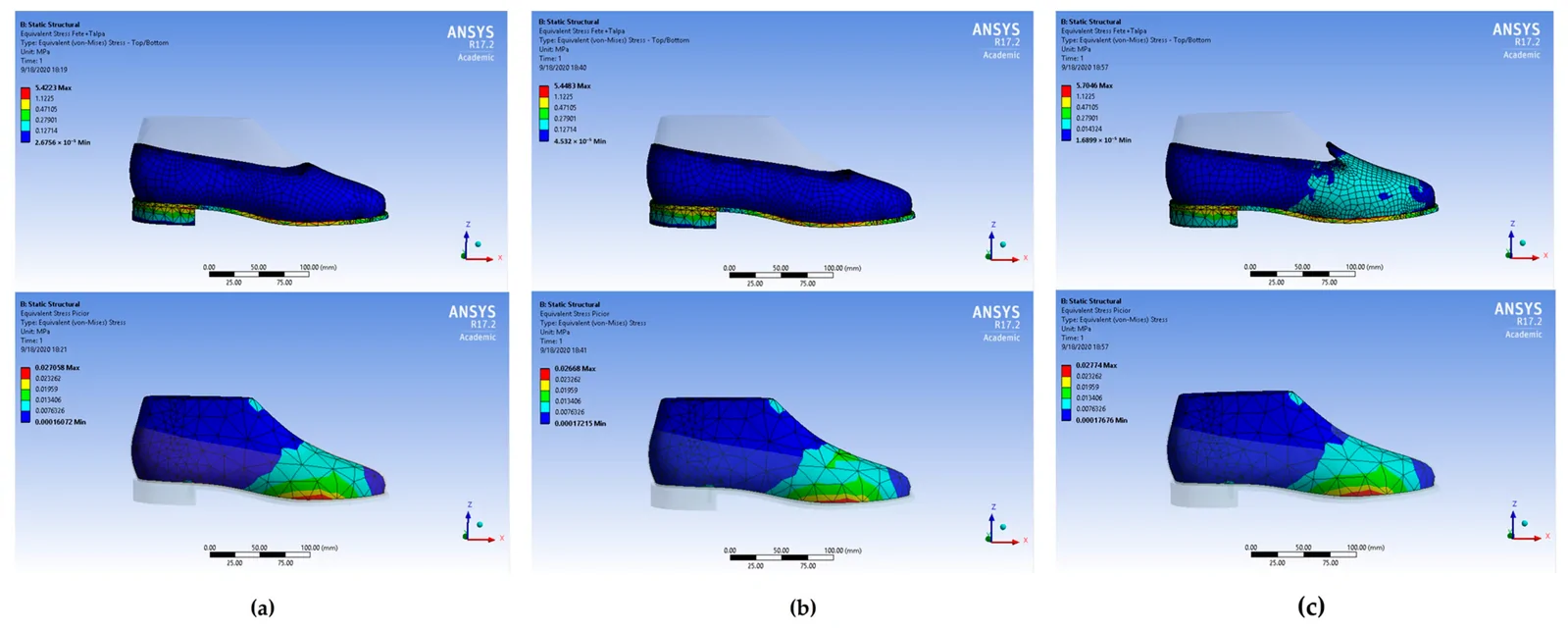
Von Mises stress distribution under loading scenario 2 for (**a**) M1, (**b**) M2, and (**c**) M3.

**Figure 10:**
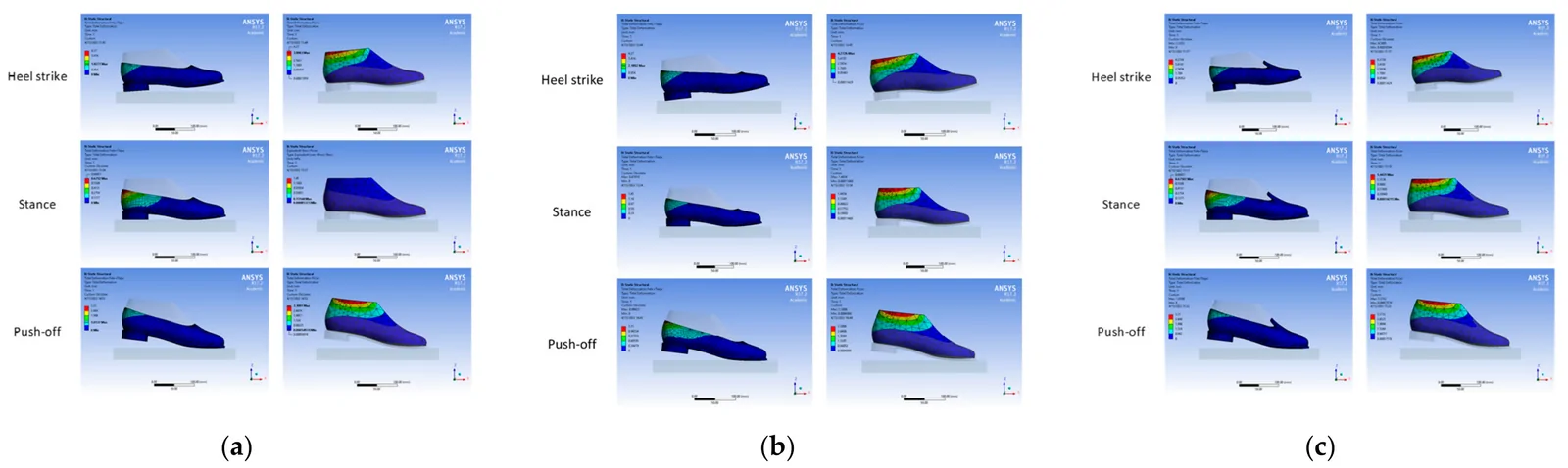
Deformation distribution under loading scenario 3 for (**a**) M1, (**b**) M2, and (**c**) M3.

**Figure 11:**
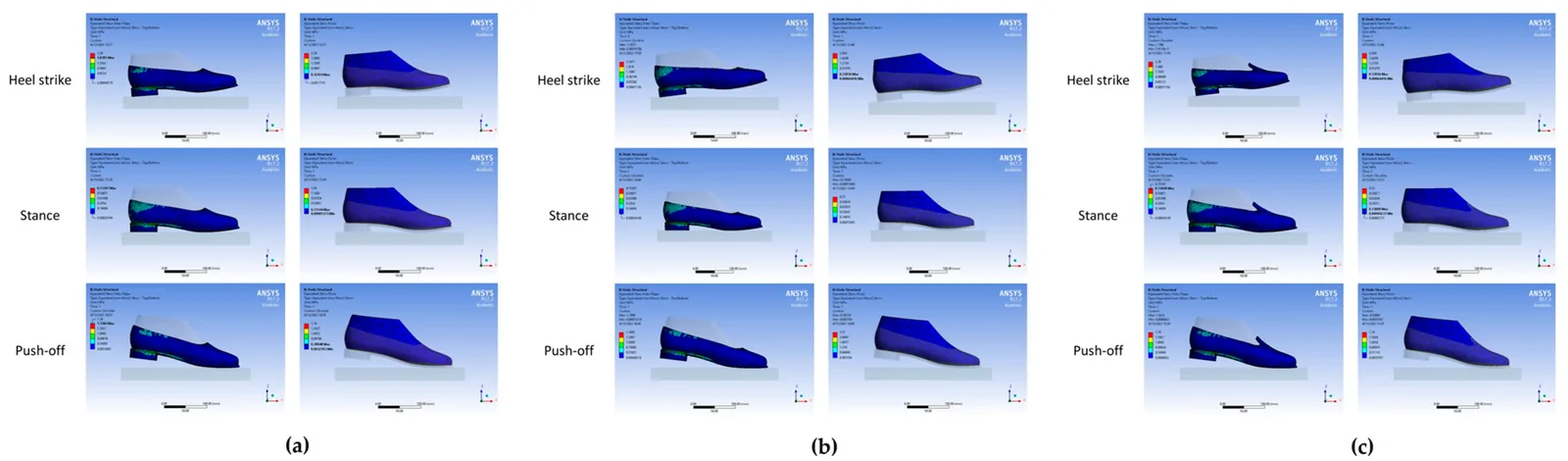
Von Mises stress distribution under loading scenario 3 for (**a**) M1, (**b**) M2, and (**c**) M3.

The software generates graphical representations which show a range of colours from blue to red, with blue indicating low values and red indicating high values of the analysed parameters. Figure 6 and Figure 7 show the deformation and von Mises distributions under loading scenario 1. Figure 8 and Figure 9 show the deformation and von Mises distributions under loading scenario 2. Figure 10 and Figure 11 show the deformation and von Mises distributions under loading scenario 3.

For each of the three models, these graphical representations, accompanied by numerical values, highlight the areas of high pressure and deformation that affect the foot and footwear during use.

**Figure 12:**
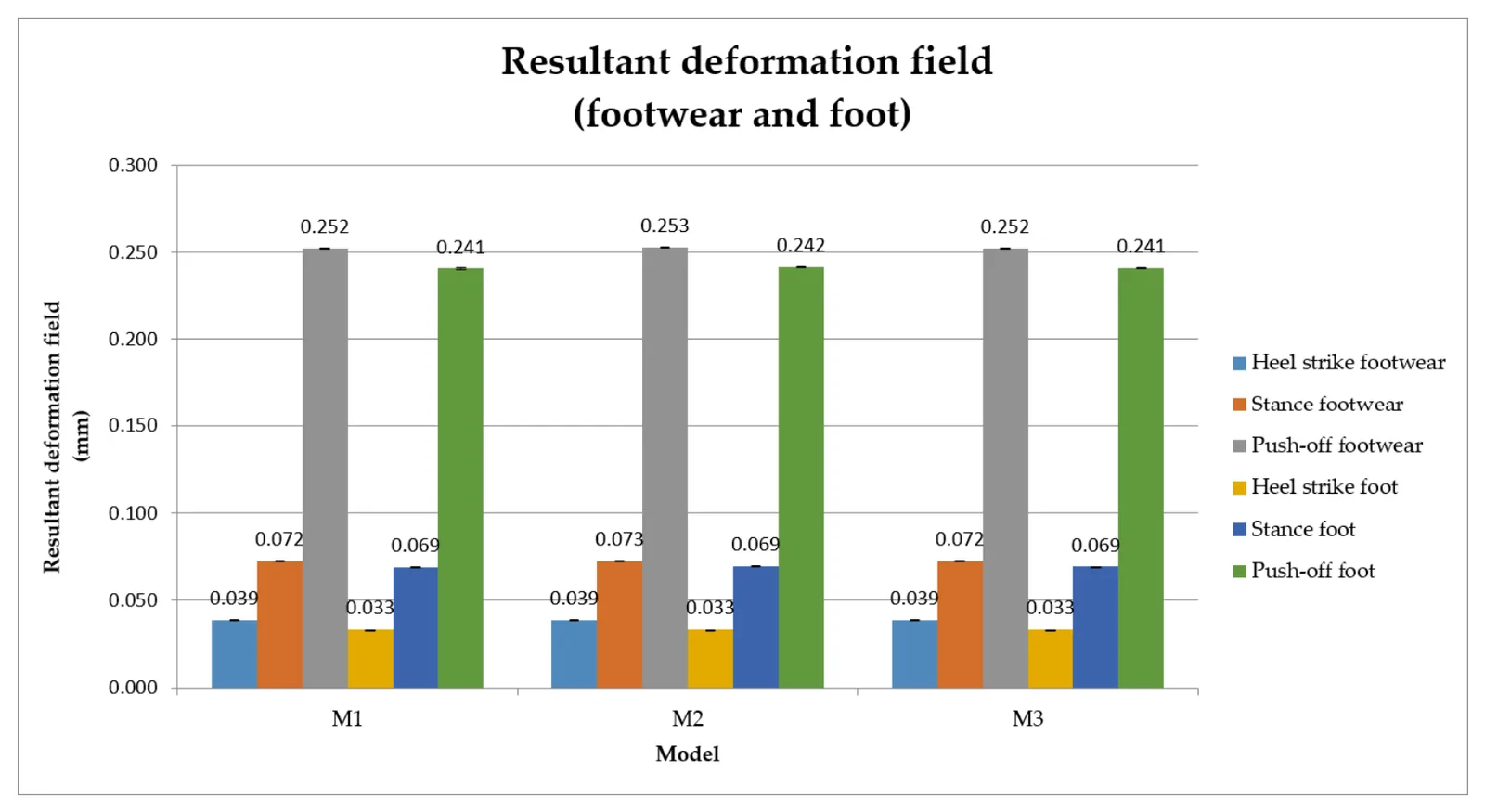
Resultant deformation field values for loading scenario 1.

**Figure 13:**
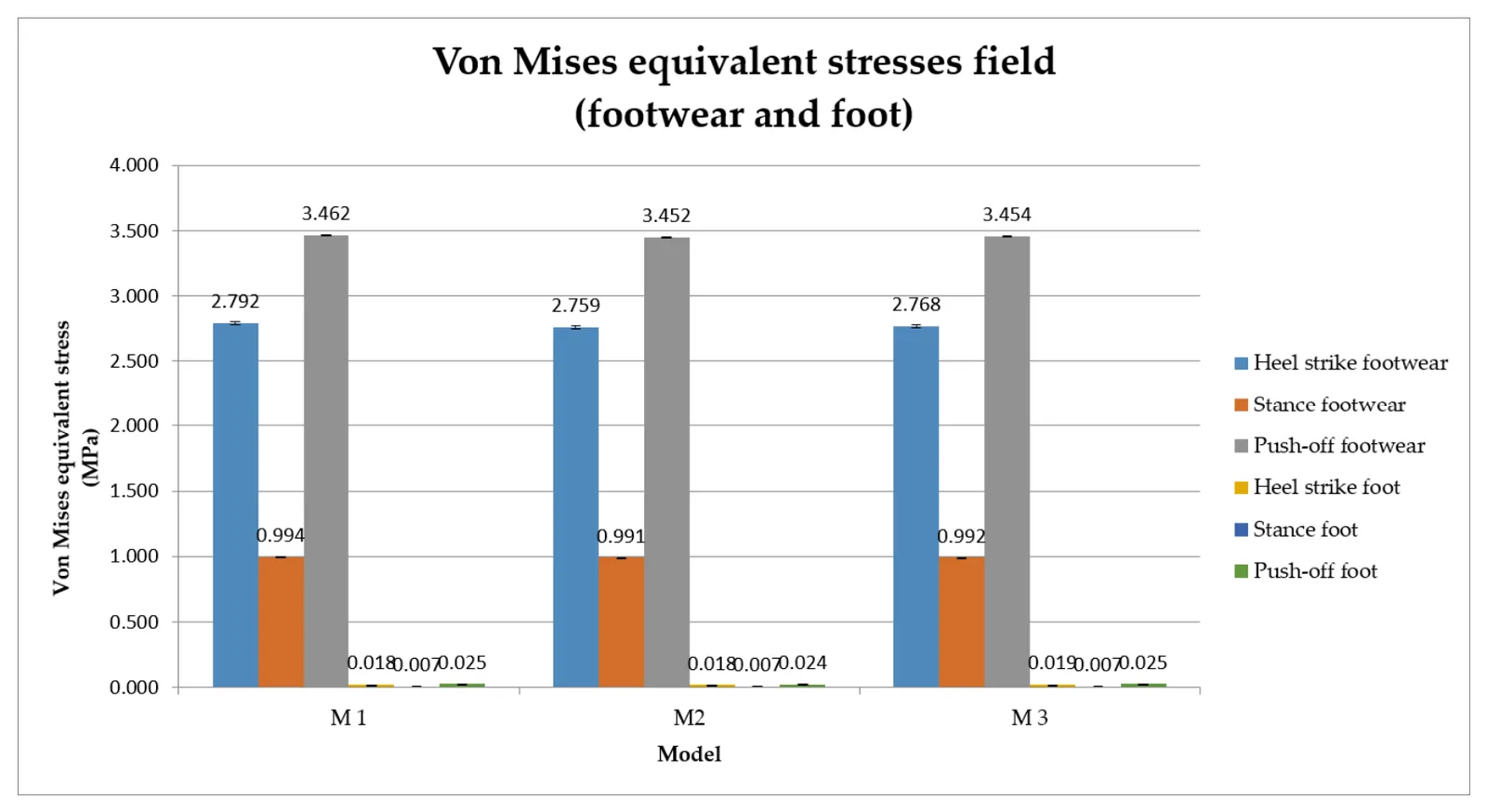
Von Mises stress field values under loading scenario 1.

Figure 12 and Figure 13 show the maximum field values of the resultant deformation and von Mises equivalent stresses for the three design variants in each gait phase under loading scenario 1.

**Figure 14:**
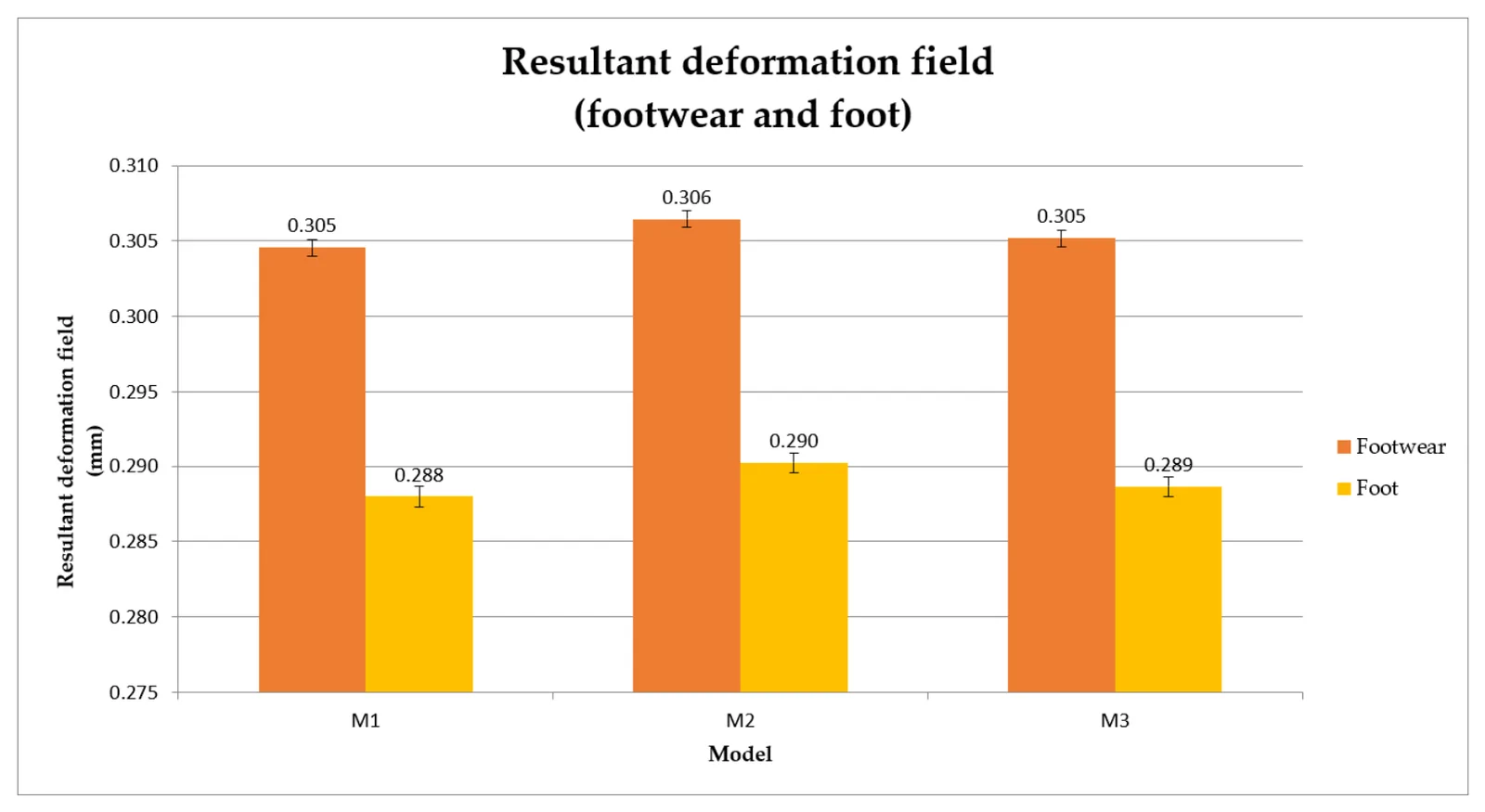
Resultant deformation field values under loading scenario 2.

**Figure 15:**
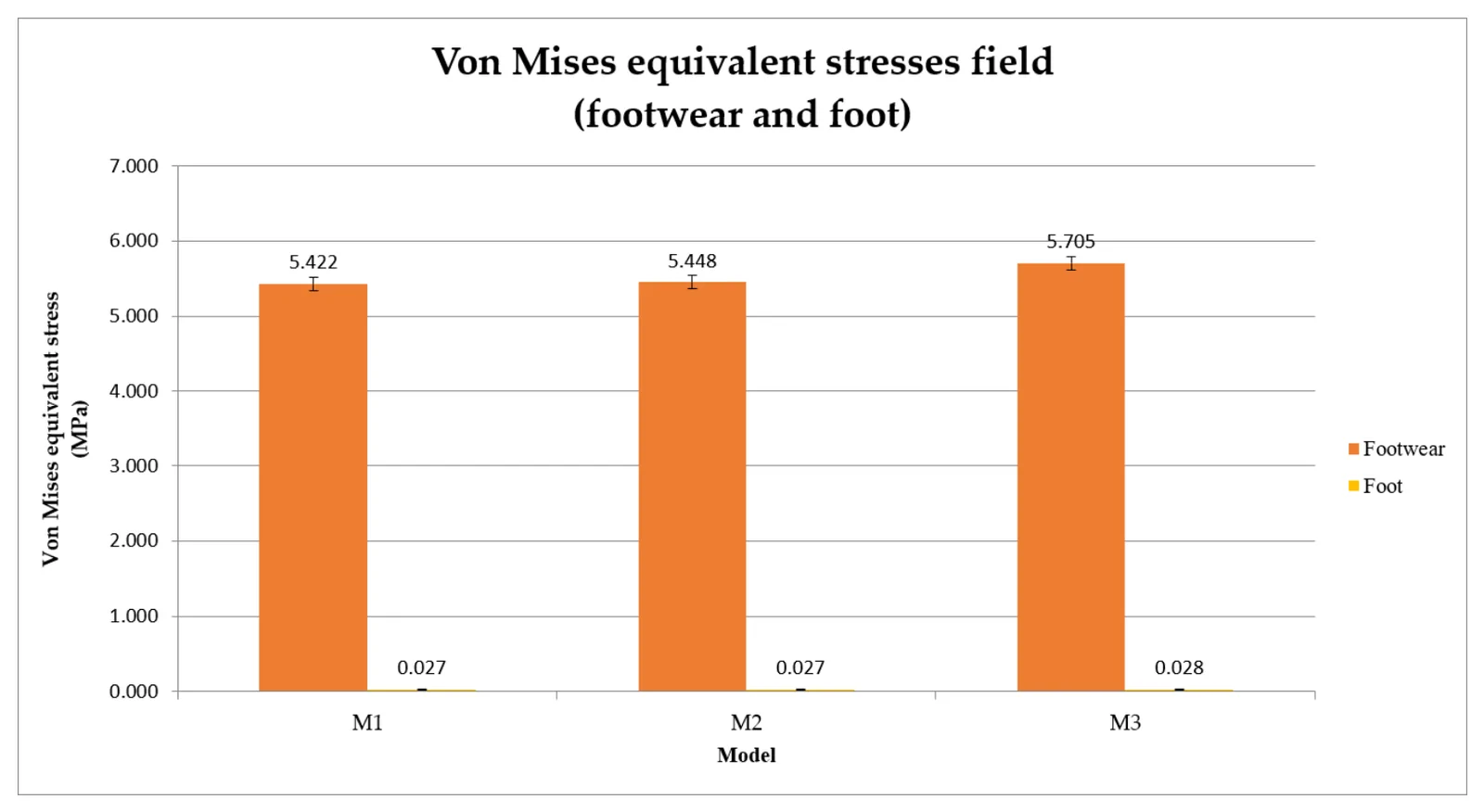
Von Mises stress field values under loading scenario 2.

Figure 14 and Figure 15 show the maximum field values of the resultant deformation and von Mises equivalent stresses for the three design variants under loading scenario 2.

**Figure 16:**
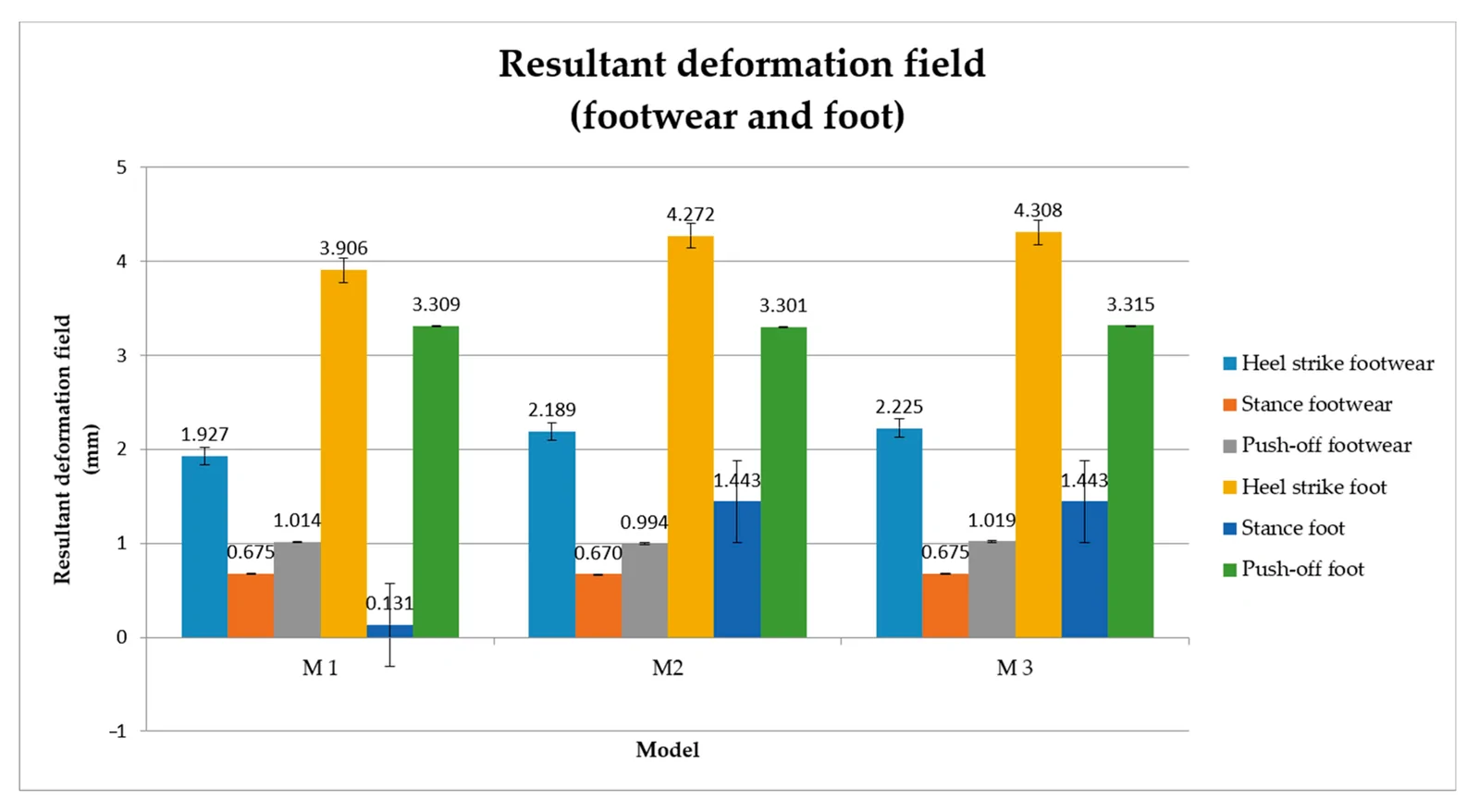
Resultant deformation field values under loading scenario 3.

**Figure 17:**
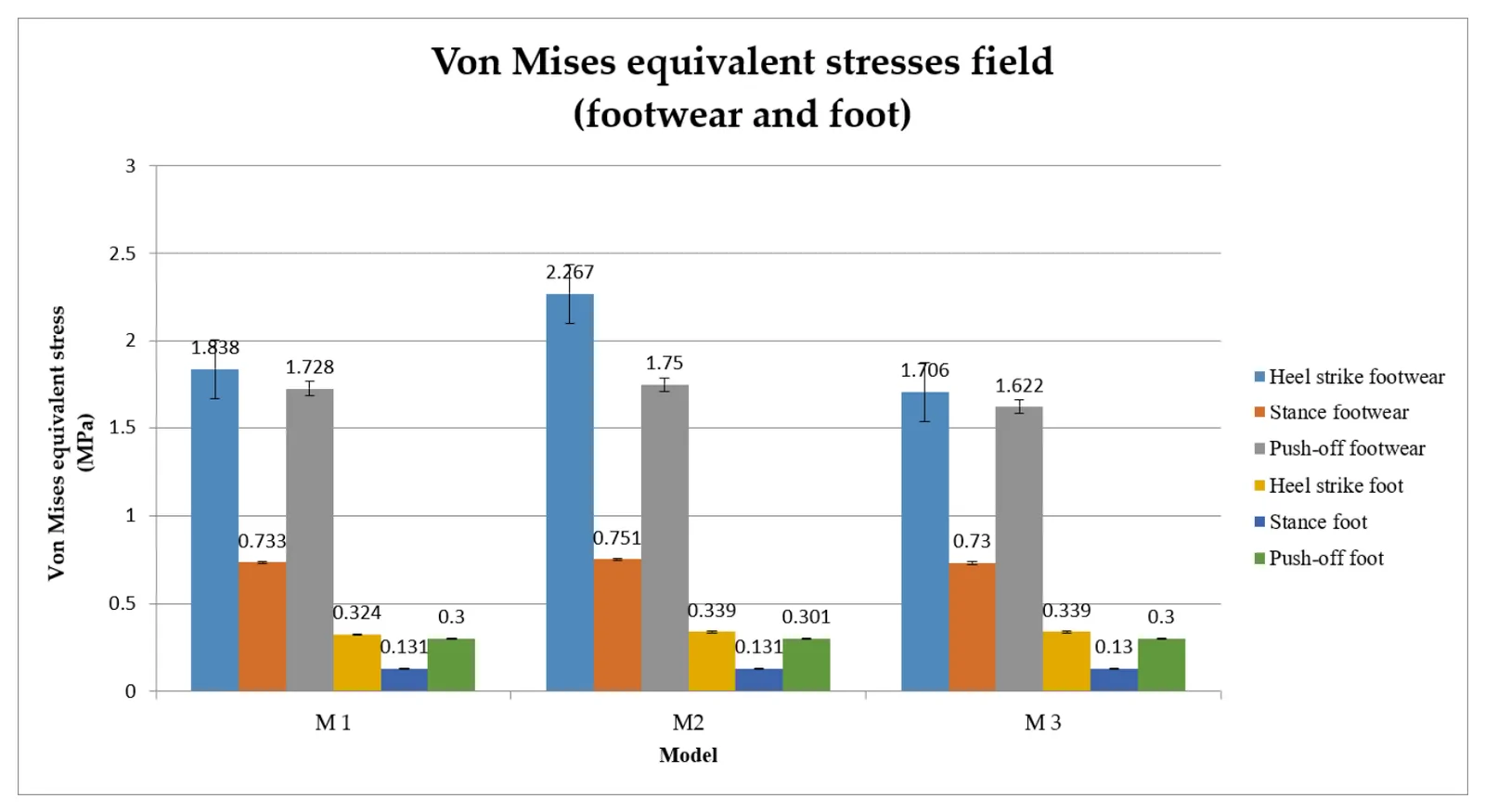
Von Mises stress field values under loading scenario 3.

Figure 16 and Figure 17 show the maximum field values of the resultant deformation and von Mises equivalent stresses for the three design variants under loading scenario 2.

The FEA simulations performed in this study produced deterministic numerical solutions for each footwear configuration under predefined material properties, boundary conditions, and loading scenarios. As each simulation generated a single solution for a given model, no repeated observations or sampling variability were available. Consequently, inferential statistical tests, which require multiple independent or repeated measurements, were not applicable. Therefore, comparisons among the three footwear configurations and the three loading scenarios were performed by directly evaluating the computed mechanical response parameters, including total deformation and von Mises stress.

Validation of the loading scenarios was carried out by comparing the values obtained by simulation with the representative mean plantar pressures resulting from the biomechanical study conducted in previous research [26].

**Figure 18:**
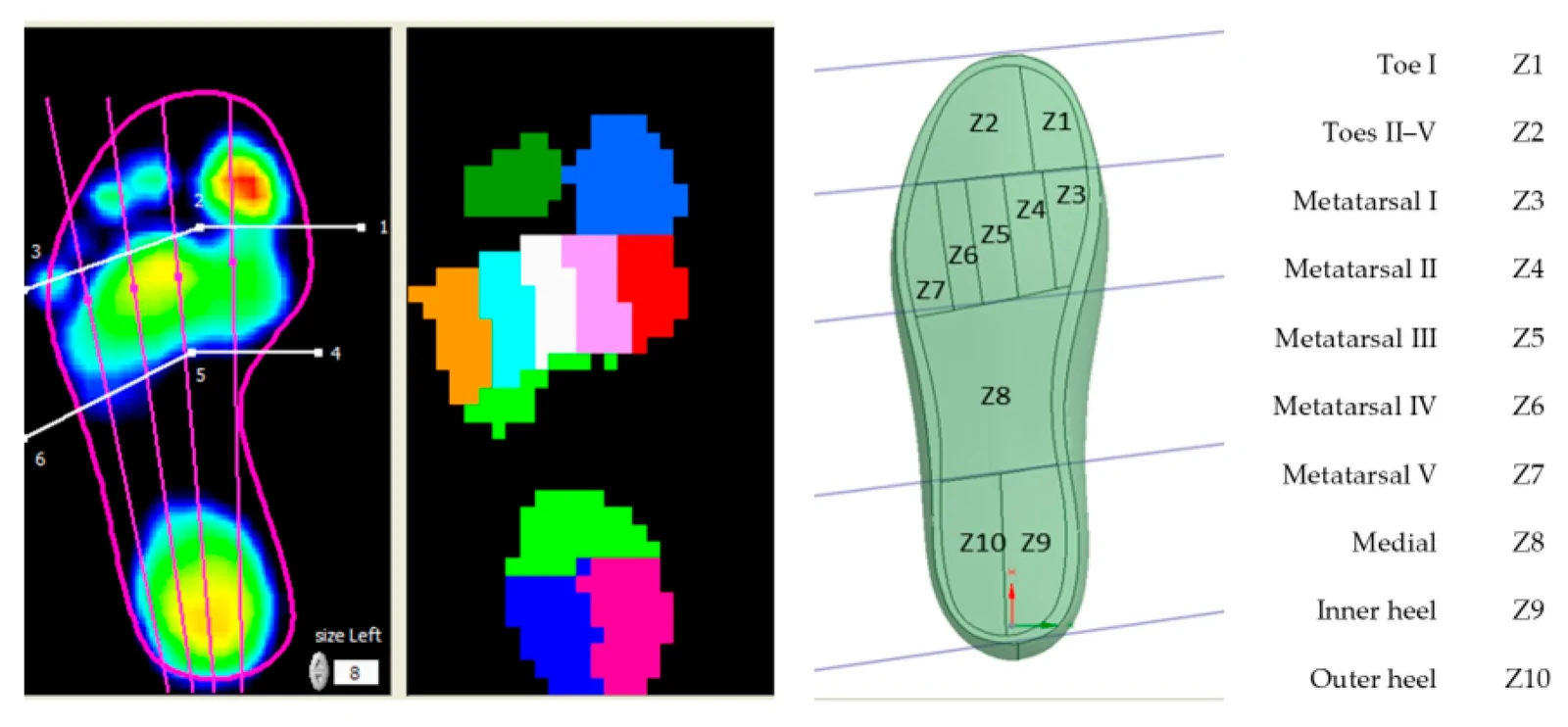
Plantar footprint in relation to foot contour.

To compare the values and validate the loading scenarios described in the Section 2, the 3D foot plantar surface was divided into ten distinct zones, similar to the Footscan system (Figure 18).

This biomechanical study found that plantar pressure values in the Z2 zone are very low and therefore insignificant for model validation. Since, in the stance phase, the pressures are distributed over the entire plantar surface, comparing them with experimental values would not be meaningful. Therefore, it was decided to compare the pressures at Z9 and Z10 during the heel strike phase and at Z1, Z3–Z7 during the push-off phase.

**Figure 19:**
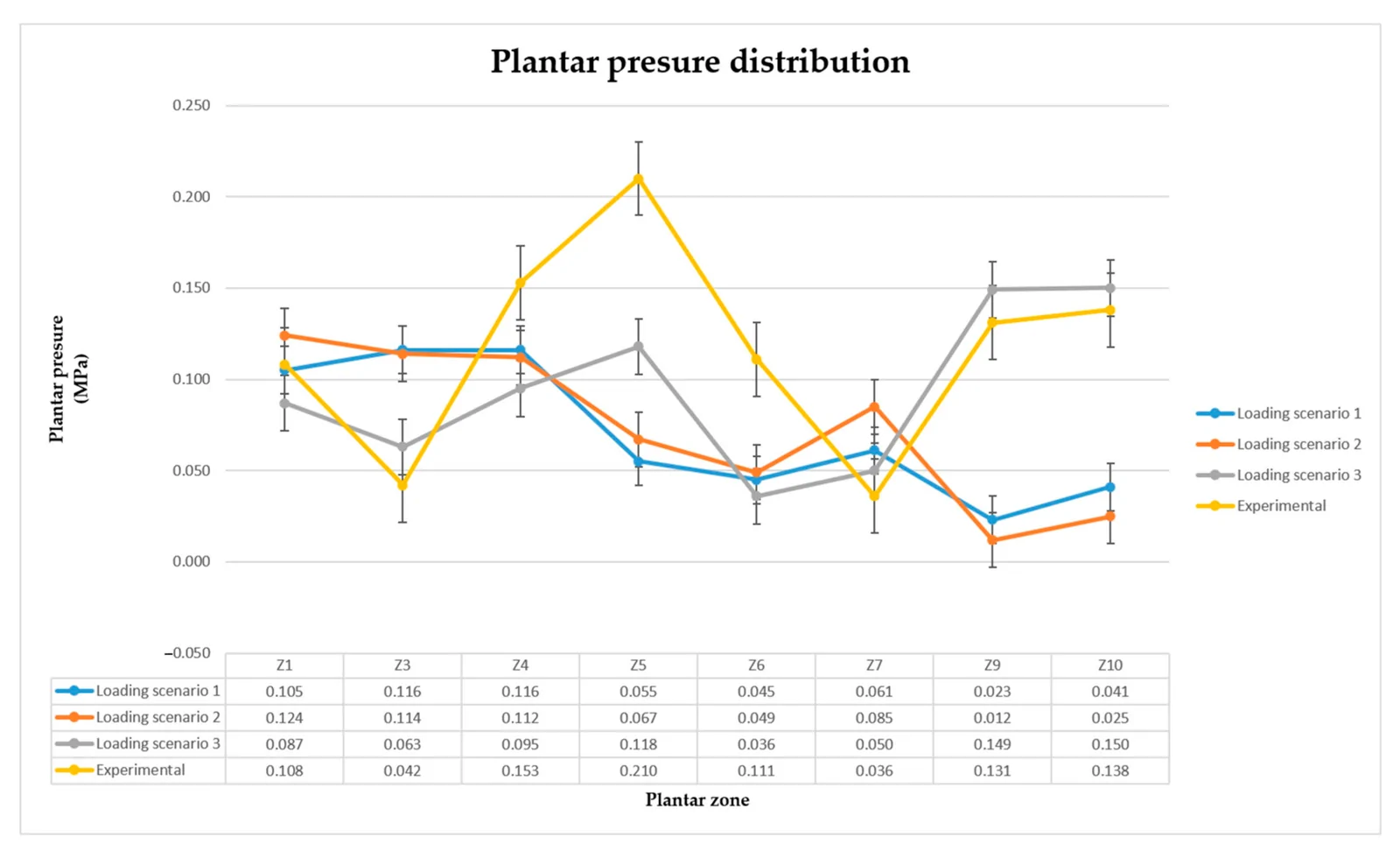
Comparative distribution of plantar pressures obtained through simulation and experimental measurements across the ten foot zones (Z1–Z10).

Figure 19 compares plantar pressures obtained via simulation and experimental measurements across the ten foot zones (Z1–Z10).

## 4. Discussion

The proposed methodology was applied to three constructive variants of low-heeled court shoes, differentiated only by the amplitude of the vamp opening, where M1 is a court shoe with a medium vamp opening, M2 is a court shoe with a wide vamp opening, and M3 is a classic court shoe with a narrow vamp opening. All three variants were developed on the same shoe last, created using mean foot dimensions derived from the anthropometric study, ensuring that any differences in mechanical behaviour among models are attributable exclusively to the vamp-opening configuration. Three loading scenarios were applied to each constructive variant, as described in Section 2. The resultant deformation field and the von Mises stress field distribution were extracted as primary output parameters for the comparative evaluation of the mechanical behaviour of the foot and footwear.

For loading scenario 1, which simulated the sequential gait phases using static representations of heel strike, stance, and push-off, the M2 model (wide vamp opening) had the greatest total deformation (0.253 mm) at push-off. This indicates that a larger vamp opening reduces upper structural stiffness and increases the displacement of the foot–footwear system when force is applied during propulsion. Similarly, these findings are consistent with existing footwear biomechanics research indicating that reduced upper constraints promote increased overall footwear flexibility and deformation during late stance and toe-off [5,6]. The M3 (narrow vamp opening) model has the lowest deformation (0.039 mm) at heel strike, suggesting that this vamp-line position provides greater stability for the foot during initial ground contact. Stress distribution analysis indicates that the M1 model produced the highest von Mises stress (3.462 MPa) at push-off due to its combination of flexibility and structural restraint, indicating localised stress concentrations. The lowest recorded stress level was observed on the M2 model during stance (0.991 MPa), indicating more uniform loading during maximum plantar contact.

The results for loading scenario 2, based on experimental force distributions across ten plantar regions, indicate small differences in resultant deformation among the three model variants, with M2 showing the highest deformation (0.307 mm) and M1 the lowest (0.305 mm). Although the differences are numerically limited, the trend remains consistent with the findings from loading scenario 1. It confirms the hypothesis that a larger vamp opening allows for better structural displacement. More noticeable differences were observed in the stress field, with M3 having the highest von Mises stress (5.700 MPa) and M1 the lowest (5.422 MPa). The higher stress concentration observed in the narrow-opening design may be explained by the increased constraint on the dorsal part of the foot, which limits material deformation and consequently transfers higher stresses to specific regions of the upper. Similar relationships between upper stiffness and stress concentration have been reported in FEA investigations of footwear structures [14,25].

The most notable differences among the various design options became evident in loading scenario 3, which concerned the angular position of the foot relative to the ground plane during heel strike, stance and push-off. In this situation, the M3 model exhibits the highest total deformation of 2.225 mm during the heel strike phase, whereas M2 shows the lowest deformation, measuring 0.670 mm during stance. The narrow vamp opening seems to limit upward movement of the foot, resulting in increased deformation of the surrounding shoe components upon impact. Meanwhile, a wider opening (M2) may contribute to better foot motion, reducing overall structural deformation during mid-stance. In terms of stress distribution, M2 shows the highest von Mises stress of 2.267 MPa during heel strike, whereas M3 has the lowest at 0.730 MPa during stance. These results suggest that although a wider opening can enhance flexibility and accommodate movement more effectively, it may also lead to localised stress concentrations upon initial contact with the ground.

The foot deformation and von Mises stress results closely follow the pattern observed for the footwear due to the strong mechanical contact between the components. In loading scenario 3, the maximum foot deformation occurred for M2 during heel strike, highlighting that the wide vamp opening provides greater freedom of movement in the forefoot and reduces dorsal foot pressure when the foot is stationary or only moderately dynamic. There is also a corresponding decrease in the amount of structural support provided to the foot at heel strike when forces are higher due to the wider vamp opening. Additionally, previous FEA and experimental studies have demonstrated that changes in shoe geometry indeed result in peak von Mises stress in the bones of the foot, with increased structural support from the heel to the forefoot. Consistent with these findings, the larger vamp opening of model M2 in the present study provided less structural resistance to the foot during the stance phase, thereby increasing foot deformation at heel strike [30,31]. Von Mises stress values in the foot were well below those associated with footwear. This finding is consistent with the established principle that properly fitting footwear absorbs and redistributes mechanical loading across the tissues of the foot.

According to the validation results, the loading scenario 3 data follow the same distribution trend of plantar pressures as in the biomechanical study. Very similar values were obtained for the toe I (Z1), metatarsal I (Z3), metatarsal V (Z7), inner (Z9), and outer heel (Z10), with differences of less than 19%. For metatarsal II (Z4), metatarsal III (Z5), and metatarsal IV (Z6), the simulation results show larger differences than the experimental values. The largest difference is observed for metatarsal IV (Z6), where the simulation value is lower than the biomechanical study value. These differences are attributable to research limitations, namely a reduced-complexity loading model and simplified foot geometry.

Despite deviations from the experimental trend in loading scenarios 1 and 2, these scenarios can still be used to make relative comparisons among the three shoe types because of the systematic offset observed across all shoe constructions. The fact that all three loading conditions produce numerically comparable and directionally consistent results further supports the overall strength of the simulation framework, even though the accuracy of each loading condition relative to experimental data was variable.

The presented FEA simulations provide useful information; however, there are a number of limitations that must be considered.

The use of a representative anthropometric foot model based on the external geometry of the foot rather than a detailed anatomical representation should be considered—although this modelling approach is appropriate for investigating the relative mechanical behaviour of different footwear upper configurations and reflects common practice in footwear engineering, it does not explicitly account for the biomechanical contribution of individual anatomical structures, such as bones, muscles, ligaments, cartilage, and plantar soft tissues. Consequently, the stress and deformation distributions obtained for the foot should be interpreted as comparative indicators of the mechanical interaction between the footwear and the representative foot model rather than as the exact physiological responses of internal tissues.

Although the quasi-static loading approach is computationally efficient, it does not include inertia or viscoelastic energy dissipation associated with dynamic gait.

Further, the assumption of linear-elastic, isotropic material properties does not accurately reflect the nonlinear and viscoelastic characteristics of real footwear components.

Additionally, boundary conditions could be enhanced to include postural and muscular activity factors that significantly affect the mechanical response of the footwear during functional tasks.

To increase the accuracy of FEA predictions, systematic mesh refinement should be performed.

Mechanical response parameters such as deformation and von Mises stress are frequently used to evaluate footwear performance, and they cannot directly quantify subjective comfort perception. Comfort is a multifactorial response influenced by mechanical, physiological, and psychological factors. Therefore, the present findings should be interpreted as indicators of mechanical performance rather than direct measures of wearer comfort.

The loading scenarios were validated by comparing simulated and experimental plantar pressures obtained from the biomechanical study based on walking trials conducted in previous research. This comparison demonstrated that the numerical model reproduced the overall trend of plantar pressure distribution observed experimentally. Nevertheless, the validation was limited to comparing pressure distribution patterns and did not include subject-specific finite element models or direct experimental measurements of footwear deformation, internal tissue stresses, or dorsal foot pressures.

## 5. Conclusions

The goal of this research was to use FEA to investigate how varying vamp amplitude affects the mechanical behaviour of women’s low-heeled court shoes and their impact on the foot.

The footwear models followed the specifications for low-heeled court shoes and were created using Delcam Crispin ShoeMaker Pro 2015 R2 software. The foot was built using a shoe last based on anthropometric data from previous research.

Material definition, 3D model editing for compatibility with the simulation application, and the establishment of boundary conditions and constraints were performed using Ansys 17.2 Academic Research Mechanical. The loading model, force distribution, force magnitudes, and constraints were configured based on three loading scenarios. The first loading scenario included body-weight forces acting as reaction forces to assess foot and footwear behaviour during the heel strike, mid-stance, and push-off phases of walking. The second loading scenario used experimentally determined force distributions across the ten defined plantar regions of the foot, derived from a biomechanical study. The third loading scenario considered the three phases of the gait cycle and the position of the foot on the support surface. The magnitudes of the forces for each gait phase were calculated using the relationships established in the first loading scenario. The resulting forces were applied to the upper surface of the foot in the vertically downward direction. This approach allowed for the evaluation of the impact of shoe design on the behaviour of both the foot and footwear and the comparison of three distinct loading scenarios.

The FEA results show that the vamp opening amplitude affects the mechanical behaviour of court shoes, influencing both deformation and stress distribution within the foot and footwear. The wider vamp opening (M2) typically allows for more flexibility and deformation than the narrower opening (M3), resulting in a more constricted structure. In addition, the medium-sized vamp opening had mixed results and, in most cases, led to the best stress distribution. The foot deformation and von Mises stress results closely follow the pattern observed for the footwear due to the strong mechanical contact between the components.

The results suggest that vamp configuration is an important consideration in shoe design and manufacturing and should be considered when optimising footwear mechanical performance. Additionally, this research provides a working methodology for footwear simulation at the design stage.

Validation of the loading scenarios was carried out by comparing simulation-derived values with the representative mean plantar pressures from a biomechanical study conducted in previous research. According to the results, the loading model 3 data follow the same distribution trend of plantar pressures as in the biomechanical study.

Although the proposed methodology provides a useful framework for evaluating the influence of vamp opening configuration on footwear biomechanics, several limitations should be acknowledged. The simplified anthropometric foot model, quasi-static loading conditions, linear-elastic material assumptions, and simplified boundary conditions limit the physiological accuracy of the simulations. Therefore, the results should be interpreted as comparative indicators of mechanical behaviour rather than direct measures of internal tissue response or wearer comfort. Future research will focus on subject-specific finite element models, dynamic gait simulations, enhanced material modelling, and experimental validation using plantar and dorsal pressure measurements, footwear deformation, and gait analysis to further improve the predictive capability of the proposed methodology.

A further direction for future research is to extend the FEA simulation framework to pathological gait conditions. Beyond normal gait, simulating gait abnormalities would provide valuable insights into footwear performance under altered biomechanical loading. Unilateral shoe wearing has been reported as one possible approach to replicating pathological motor patterns and could serve as a basis for future computational and experimental investigations [32,33,34].

Additionally, examining the upper assembly—including the lining layers and how the mechanical behaviour of the two materials overlaps—is a viable means of expanding the predictive capability of computational models of footwear.

## Figures and Tables

**Table 1:** Summary of reviewed finite element studies in footwear.

Research Focus	Main Contribution	Identified Limitations	Reference
Systematic review of footwear FEA	Summarised modelling approaches, material models, loading conditions and validation methods used in footwear biomechanics.	Most studies focused on the foot–sole–ground interaction; limited investigation of upper design parameters.	Song et al. (2022) [6]
Midsole hardness	Investigated the influence of midsole stiffness on plantar fascia stress and outsole contact pressure using a coupled foot–shoe model.	Focus restricted to the sole system; upper geometry not considered.	Zhu et al. (2023) [7]
Carbon-fibre plate position and thickness	Demonstrated that lower-positioned and thicker carbon-fibre plates improved forefoot load distribution and reduced metatarsal loading.	Upper construction was not investigated.	Song et al. (2023) [8]
TPU midsole	Demonstrated pressure reduction for diabetic footwear using auxetic lattice midsoles validated with plantar pressure measurements.	Upper design parameters were not analysed.	Zhang et al. (2024) [10]
Midsole optimisation	Used Taguchi optimisation to identify optimal heel cup geometry, insole material and midsole thickness for plantar pressure redistribution.	Focused exclusively on sole components.	Yang et al. (2022) [11]
Outsole contact angle	Investigated heel–ground contact angle and its effect on metatarsal stress during running.	Upper geometry was not included.	Zhou et al. (2024) [12]
Footwear upper deformation	Developed the first FEA model describing upper deformation during gait.	Simplified linear-elastic material representation.	Ruperez et al. (2008) [13]
Upper material modelling	Proposed an orthotropic leather model accounting for large deformations and validated it experimentally.	Focused on material behaviour rather than constructive design.	Ruperez et al. (2012) [14]
Upper material selection	Compared different upper materials under gait loading and demonstrated their influence on footwear deformation and stress distribution.	Upper geometry remained unchanged.	Seul et al. (2023) [15]
Pattern lines and stitching	Evaluated the influence of pattern line placement and stitch number on footwear performance during gait.	Investigated manufacturing parameters rather than overall upper geometry.	Seul et al. (2025) [16]
Vamp opening configuration of women’s court shoes	Investigates the biomechanical influence of three vamp opening configurations using three loading scenarios and validates the loading methodology against experimental plantar pressure data.	Provides evidence regarding the effect of upper geometry on footwear and foot biomechanics.	Present study

**Table 2:** Anthropometric dataset [17].

No.	Code	L_f_ (mm)	G_b_ (mm)	B_f_ (mm)	C_i_ (mm)	A_tI_ (o)	A_tV_ (o)	H_tI_ (mm)	H_tV_ (mm)	Foot Size (INFOOT)
1	S7_36	222.7	241.1	95.9	235.0	10.9	8.8	21.4	21.9	36.0
2	S2_36	226.1	226.3	89.8	223.6	3.8	11.7	20.7	19.1	36.0
3	S9_36	226.3	217.0	86.8	215.9	3.6	12.8	19.6	15.1	36.0
4	S6_36	228.5	223.2	90.8	224.1	11.2	−0.5	18.5	17.7	36.5
5	S5_36	228.7	220.0	89.2	219.8	7.1	12.9	20.1	15.6	36.5
6	S10_36	228.8	229.3	93.8	221.1	7.2	15.5	20.7	19.2	36.5
7	S3_36	232.5	222.1	93.6	215.6	4.9	7.7	19.1	15.9	36.5
8	S4_36	233.9	216.0	90.8	174.6	13.8	9.0	18.5	16.0	37.5
9	S8_37	233.9	216.0	90.8	174.6	13.8	9.0	18.5	16.0	37.5
10	S9_37	234.2	218.2	88.8	217.2	2.8	10.8	19.0	17.4	37.5
11	S1_36	235.2	235.0	96.6	227.8	7.0	10.2	21.2	17.6	37.5
12	S13_37	236.3	237.9	98.1	238.2	10.4	9.0	20.6	19.3	37.5
13	S11_37	237.2	228.8	92.3	228.3	15.3	5.1	21.7	17.8	37.5
14	S6_38	237.3	240.6	94.7	238.9	5.4	9.4	20.7	20.2	37.5
15	S10_37	238.0	208.8	84.5	219.0	7.4	7.9	17.4	14.4	37.5
16	S7_38	238.2	220.0	90.1	220.5	4.9	10.0	20.2	16.1	37.5
17	S7_37	238.3	233.2	94.6	229.1	−7.6	15.4	21.2	19.3	37.5
18	S1_37	238.6	228.2	93.5	221.8	4.6	10.9	20.6	19.4	38.0
19	S2_38	238.6	228.2	93.5	221.8	4.6	10.9	20.6	19.4	38.0
20	S4_38	239.1	239.7	99.1	247.1	10.8	18.3	23.2	20.4	38.0
21	S3_38	239.2	231.1	94.9	223.2	14.8	9.3	20.9	17.1	38.0
22	S9_38	239.5	238.9	97.2	234.7	1.3	11.3	21.2	21.9	38.0
23	S5_37	240.0	248.3	100.0	244.4	5.6	17.2	22.4	22.3	38.0
24	S4_37	240.5	219.5	88.7	214.0	4.6	5.3	20.2	15.7	38.0
25	S3_37	241.3	222.6	89.9	223.9	6.1	3.1	20.0	16.5	38.0
26	S8_36	241.8	225.9	93.0	223.3	6.4	19.5	19.7	16.8	38.0
27	S2_37	242.3	227.4	94.7	235.1	4.9	13.6	17.6	17.6	38.0
29	S12_37	243.2	238.3	98.2	238.6	7.6	5.2	22.2	19.9	38.5
30	S6_37	244.1	230.9	95.4	220.5	7.3	7.2	21.3	17.4	38.5
31	S1_38	244.6	245.8	97.8	253.7	7.3	6.0	22.2	19.9	38.5
32	S5_38	249.1	227.5	92.9	182.1	5.2	8.0	20.4	18.6	38.5

**Table 4:** Value distribution for foot length [17].

Class Range (mm)		Centre of the Class	Absolute Frequency	Relative Frequency (%)
222.5	227.5	225	3	9.7
227.5	232.5	230	4	12.9
232.5	237.5	235	7	22.6
237.5	242.5	240	13	41.9
242.5	247.5	245	3	9.7
247.5	252.5	250	1	3.2

**Table 5:** Value distribution for ball girth circumference [17].

Class Range (mm)		Centre of the Class	Absolute Frequency	Relative Frequency (%)
208.8	215.4	212.1	1	3.2
215.4	222.0	218.7	7	22.6
222.0	228.5	225.2	9	29.0
228.5	235.1	231.8	6	19.4
235.1	241.7	238.4	6	19.4
241.7	248.3	245.0	2	6.5

**Table 6:** Properties of the materials used in the analysis.

	**Simplified Foot Structure**	**Leather (Uppers)**
Young’s modulus (MPa)	4.47	20
Poisson coefficient	0.45	0.4
	**Polyurethane (Insole)**	**Rubber (Sole)**
Young’s modulus (MPa)	60	1000
Poisson coefficient	0.4	0.42
	**Concrete (Support)**	
Young’s modulus (MPa)	90,000	
Poisson coefficient	0.18	

**Table 7:** Mean force values applied as loading conditions for the simulation of normal gait.

Force (N)	Contact Time (ms)
141.9	612.4
19.0	504.9
40.8	501.1
142.9	585.8
163.2	587.2
92.2	575.5
34.0	488.4
34.0	199.0
184.4	173.1
170.8	178.5

**Table 8:** Maximum resultant deformation values extracted from the finite element simulations for each model under loading scenario 1.

Code	Phase	Footwear and Foot Maximum Value [mm]	Footwear Maximum Value [mm]	Foot Maximum Value [mm]
M1	Heel strike	0.039	0.039	0.033
	Stance	0.072	0.072	0.069
	Push-off	0.252	0.252	0.241
M2	Heel strike	0.039	0.039	0.033
	Stance	0.073	0.073	0.069
	Push-off	0.253	0.253	0.242
M3	Heel strike	0.039	0.039	0.033
	Stance	0.072	0.072	0.069
	Push-off	0.252	0.252	0.241

**Table 9:** Maximum von Mises stress field values extracted from the finite element simulations for each model under loading scenario 1.

Code	Phase	Footwear and Foot Maximum Value [MPa]	Footwear Maximum Value [MPa]	Foot Maximum Value [MPa]
M1	Heel strike	2.792	2.792	0.018
	Stance	0.994	0.994	0.007
	Push-off	3.462	3.462	0.025
M2	Heel strike	2.759	2.759	0.018
	Stance	0.991	0.991	0.007
	Push-off	3.452	3.452	0.024
M3	Heel strike	2.768	2.768	0.019
	Stance	0.992	0.992	0.007
	Push-off	3.454	3.454	0.025

**Table 10:** Maximum resultant deformation values extracted from the finite element simulations for each model under loading scenario 2.

Code	Footwear and Foot Maximum Value [mm]	Footwear Maximum Value [mm]	Foot Maximum Value [mm]
M1	0.305	0.305	0.288
M2	0.306	0.306	0.290
M3	0.305	0.305	0.289

**Table 11:** Maximum von Mises stress field values extracted from the finite element simulations for each model under loading scenario 2.

Code	Footwear and Foot Maximum Value [MPa]	Footwear Maximum Value [MPa]	Foot Maximum Value [MPa]
M1	5.422	5.422	0.027
M2	5.448	5.448	0.027
M3	5.705	5.705	0.028

**Table 12:** Maximum resultant deformation values extracted from the finite element simulations for each model under loading scenario 3.

Code	Phase	Footwear and Foot Maximum Value [mm]	Footwear Maximum Value [mm]	Foot Maximum Value [mm]
M1	Heel strike	3.906	1.927	3.906
	Stance	1.453	0.675	0.131
	Push-off	3.309	1.014	3.309
M2	Heel strike	4.272	2.189	4.272
	Stance	1.443	0.670	1.443
	Push-off	3.301	0.994	3.301
M3	Heel strike	4.308	2.225	4.308
	Stance	1.443	0.675	1.443
	Push-off	3.315	1.019	3.315

**Table 13:** Maximum von Mises stress field values extracted from the finite element simulations for each model under loading scenario 3.

Code	Phase	Footwear and Foot Maximum Value [MPa]	Footwear Maximum Value [MPa]	Foot Maximum Value [MPa]
M1	Heel strike	1.838	1.838	0.324
	Stance	0.733	0.733	0.131
	Push-off	1.728	1.728	0.300
M2	Heel strike	2.267	2.267	0.339
	Stance	0.751	0.751	0.131
	Push-off	1.750	1.750	0.301
M3	Heel strike	1.706	1.706	0.339
	Stance	0.730	0.730	0.130
	Push-off	1.622	1.622	0.300

## Data Availability

The raw data supporting the conclusions of this article will be made available by the authors on request.
